# Non-canonical chemical feedback self-limits nitric oxide-cyclic GMP signaling in health and disease

**DOI:** 10.1038/s41598-020-66639-w

**Published:** 2020-06-19

**Authors:** Vu Thao-Vi Dao, Mahmoud H. Elbatreek, Martin Deile, Pavel I. Nedvetsky, Andreas Güldner, César Ibarra-Alvarado, Axel Gödecke, Harald H. H. W. Schmidt

**Affiliations:** 1 Department of Pharmacology and Personalised Medicine, MeHNS, FHML, Maastricht University, Maastricht, The Netherlands; 2 Department of Psychiatry, Psychosomatic Medicine and Psychotherapy, University Hospital Frankfurt, Frankfurt, Germany; 3 Department for Pharmacology and Toxicology, Faculty of Pharmacy, Zagazig University, Zagazig, Egypt; 4 Primary Care Center, Altenberger Str. 27, 01277, Dresden, Germany; 5 Universitätsklinikum Münster, Medical Clinic D, Medical Cell Biology, Münster, Germany; 6 Residency Anesthesiology, Department of Anesthesiology and Critical Care Medicine, Technische Universität, Dresden, Germany; 7 Facultad de Química, Universidad Autónoma de Querétaro, Santiago de Querétaro, Mexico; 8 Institut für Herz- und Kreislaufphysiologie Heinrich-Heine-Universität, Düsseldorf, Germany

**Keywords:** Pharmacology, Diseases

## Abstract

Nitric oxide (NO)-cyclic GMP (cGMP) signaling is a vasoprotective pathway therapeutically targeted, for example, in pulmonary hypertension. Its dysregulation in disease is incompletely understood. Here we show in pulmonary artery endothelial cells that feedback inhibition by NO of the NO receptor, the cGMP forming soluble guanylate cyclase (sGC), may contribute to this. Both endogenous NO from endothelial NO synthase and exogenous NO from NO donor compounds decreased sGC protein and activity. This effect was not mediated by cGMP as the NO-independent sGC stimulator, or direct activation of cGMP-dependent protein kinase did not mimic it. Thiol-sensitive mechanisms were also not involved as the thiol-reducing agent N-acetyl-L-cysteine did not prevent this feedback. Instead, both *in-vitro* and *in-vivo* and in health and acute respiratory lung disease, chronically elevated NO led to the inactivation and degradation of sGC while leaving the heme-free isoform, apo-sGC, intact or even increasing its levels. Thus, NO regulates sGC in a bimodal manner, acutely stimulating and chronically inhibiting, as part of self-limiting direct feedback that is cGMP independent. In high NO disease conditions, this is aggravated but can be functionally recovered in a mechanism-based manner by apo-sGC activators that re-establish cGMP formation.

## Introduction

The nitric oxide (NO)-cGMP signaling pathway plays several essential roles in physiology, including cardiopulmonary homeostasis^1,2^. The main receptor and mediator of NO’s actions is soluble guanylate cyclase (sGC), a heterodimeric heme protein. In its Fe(II)heme-containing state, sGC binds NO and is thereby activated to convert GTP to the second messenger, cGMP, whose steady-state levels are counter-regulated by different phosphodiesterases (PDEs)^3^. cGMP exerts its cardiopulmonary effects via activating cGMP-dependent protein kinase I (PKG)^4^. The latter results in protective vasodilation, anti-proliferation, and anti-thrombosis^5^. In disease, heme loss and the appearance of NO-insensitive apo-sGC impair NO-cGMP signaling^6,7^.

In addition to acutely activating sGC, NO appears to have further roles in sGC regulation. During enzyme maturation, NO facilitates heme incorporation into apo-sGC^8,9^, and activation of sGC by NO is followed by acute and rapid desensitization involving protein S-nitrosylation^10,11^. In addition, chronic exposure to NO donor drugs has been suggested to negatively affect sGC activity in a not fully reversible manner^12–14^. It is unclear, however, whether this pharmacological effect also pertains to endogenously formed NO and has pathophysiological relevance.

Here, we examine this important knowledge gap in the (patho)biology of NO. As model systems, we chose porcine pulmonary artery endothelial cells (PPAECs) as they relate to the clinical application of NO and cGMP-modulating drugs in pulmonary hypertension^15,16^. We investigate the effects of chronic exposure to exogenous (from NO donor drugs) and endogenous NO on sGC protein and activity in these cells. In addition, we investigate in health and disease, whether chronic effects of NO on sGC involve canonical cGMP signaling, thiol modulation, or formation of heme-free sGC (apo-sGC). As disease model, we use again a condition related to pulmonary hypertension and chronically elevated levels of NO, i.e., porcine acute respiratory disease syndrome (ARDS)^17–19^.

## Results

### NO chronically decreases vascular sGC protein and activity *in-vivo* and *in-vitro*

**Figure 1:**
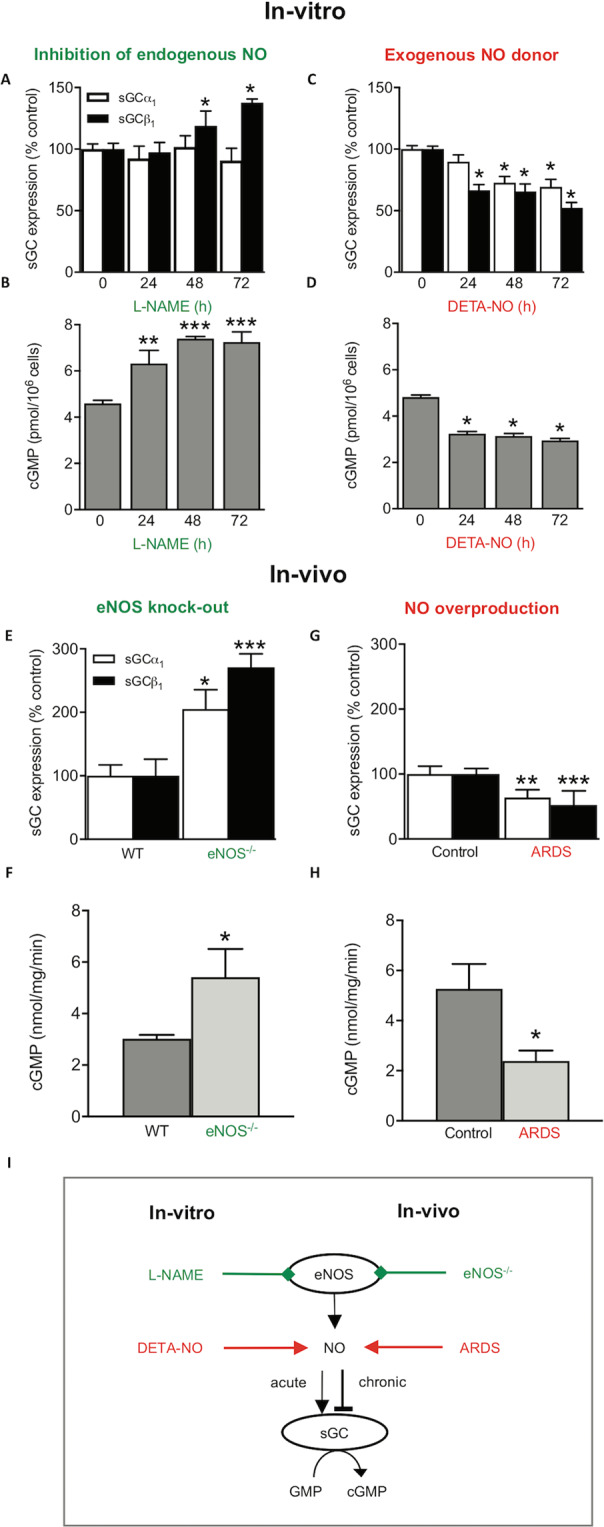
Chronic NO decreases vascular sGC protein and activity *in-vivo* and *in-vitro*. (**A**) Inhibiting basal NO formation in PPAECs by L-NAME (100 μM) for up to 72 h increased sGCβ_1_ expression (N = 6). **(B)** This upregulation was associated with increased sGC activity (N = 3). Exposing cells to supra-physiological levels of NO by chronic exposure to the NO donor compound, DETA/NO (100 µM), for up to 72 h decreased both sGCα_1_ and sGCβ_1_ protein **(C)** (N = 6) and sGC activity **(D)** (N = 5). *In-vivo* validation of the *in-vitro* observations showed in eNOS knockout mice (eNOS^−/−^) mice increased sGC protein **(E)** and activity levels **(F)** (N = 9), and in a porcine lung disease model (ARDS) characterized by NO overproduction, decreased sGCα_1_ and sGCβ_1_ protein **(G)** (N = 5) and sGC activity levels **(H)** (N = 3). Data are expressed as mean ± SEM. *,**,***p < 0.05, 0.01 or 0.001 *vs*. control, respectively. **(I)** A schematic summary showing that both *in-vitro* (porcine lung endothelial cells) and *in-vivo* (the porcine lung disease model, ARDS) both endogenous and exogenous NO downregulate sGC protein and activity. Representative full-length blots are presented in Supplementary Figure S4.

For analyzing the chronic effects of NO at a mechanistic level, PPAECs were incubated for up to 72 h in the presence of the NO synthase (NOS) inhibitor, N^G^-nitro L-arginine methyl ester (L-NAME), and sGC expression and activity were measured. In the presence of L-NAME to eliminate endogenous NO formation, protein levels of the heme-binding sGCβ_1_ subunit were increased (Fig. 1A), and this was also associated with increased sGC activity (Fig. 1B). Next, we tested the reverse, i.e., whether an increase of NO to supra-physiological concentrations^20–22^ by chronic exposure to the long-acting NO donor compound, DETA/NO, downregulates sGC. When establishing the concentration-dependence of DETA/NO on sGC expression, we found 100 µM to exert a maximal downregulation (Supplementary Fig. S1) without affecting cell viability. In line with this, DETA/NO was also used in previous studies at a concentration of 100 µM to mimic chronically high-NO disease conditions^20–22^. Therefore, in all subsequent experiments, cells were exposed to 100 µM DETA/NO, unless otherwise indicated. Pre-incubating PPAECs with DETA/NO (100 µM) decreased both sGCα_1_ and sGCβ_1_ protein (Fig. 1C) and sGC activity (Fig. 1D). Thus, *in-vitro* in PPAECs, endogenous NO chronically downregulates sGC protein and activity in an L-NAME-reversible manner, and this is further aggravated by exogenous, pharmacologically applied NO in supra-physiological concentrations.

Next, we wanted to validate these *in-vitro* observations at an *in-vivo* level. To eliminate endogenous NO formation similar to the *in-vitro* L-NAME experiment, we chose eNOS knockout mice (eNOS^−/−^); as a high-NO condition, the previously extensively validated porcine ARDS model^17,19,23^. In line with our observations in PPAECs, eNOS^−/−^ mice showed increased protein levels of sGCα_1_ and sGCβ_1_ (Fig. 1E) and increased sGC-activity (Fig. 1F). In the high-NO porcine ARDS model, sGCα_1_ and sGCβ_1_ protein levels (Fig. 1G) and sGC activity were decreased (Fig. 1H). These data collectively suggest in both *in-vitro* and *in-vivo* that decreasing endogenous NO elevates, and increasing it lowers sGC protein subunit levels and sGC activity (Fig. 1I), respectively.

### cGMP/PKG does not mediate the downregulation of sGC protein and activity by chronic NO

**Figure 2:**
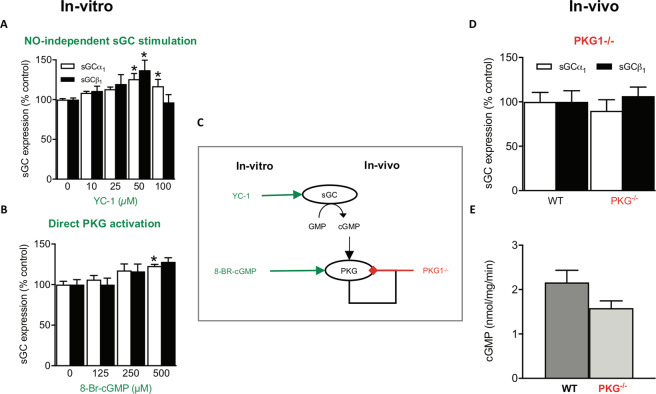
PKG does not mediate the downregulation of sGC protein and activity by chronic NO. When PPAECs were incubated for 72 h in the absence or presence of increasing concentrations of **(A)** the NO-independent sGC stimulator, YC-1 (N = 6), this did not cause downregulation of sGCα_1_ and sGCβ_1_ expression but rather a small upregulation. Consistent with this, in **(B)** the direct PKG activator 8-Br-cGMP (N = 6) led to increased sGCα_1_ protein expression. **(C)** The scheme summarizes the *in-vivo* and *in-vitro* data suggesting that the downregulation of sGC protein and activity by chronic NO is cGMP- and PKG-independent and thus appeared to be due to a non-canonical mechanism. **(D)** sGC protein expression (N = 4) and **(E)** activity (N = 4) are not altered in PKG^−/−^ as compared to wildtype mice. Data are expressed as mean ± SEM. *,**,***p < 0.05, 0.01 or 0.001 *vs*. control, respectively. Representative full-length blots are presented in Supplementary Figure S4.

Next, we aimed to clarify the mechanisms underlying the downregulation of sGC protein and activity by chronic NO. First, we tested whether cGMP/PKG signaling is involved, as it had been shown previously to decrease both sGC activity^24^ and expression^25^. Of experimental importance, _c_ell passaging can cause downregulation of PKG and prevent the detection of its-dependent signaling^26–29^. Hence, we, therefore, restricted our studies to low passage number cells and ensured fully functional PKG signaling by validating the known autoregulation of PKG expression^30,31^. Indeed, in our PPAEC system, both the PKG activator, 8-Br-cGMP, and the NO-independent sGC stimulator and PDE inhibitor, YC-1^32^, were able to reproduce the reduction of PKG expression (Supplementary Fig. S2) confirming the presence of a fully functional PKG. We then studied whether the observed downregulation of sGC protein and activity by NO can be mimicked by cGMP or is prevented by inhibiting PKG. When we exposed PPAECs, however, for 72 h to different concentrations of the sGC stimulator and PDE inhibitor, YC-1, to raise cGMP in a NO-independent manner, or to the direct PKG activator, 8-Br-cGMP, neither sGC protein nor activity were lowered (*cf*. to Fig. 1). Unexpectedly, we observed even a slight upregulation of sGC protein (Fig. 2A,B). Consistent with this, the NO-induced downregulation of sGC could not be prevented by co-incubation with the PKG inhibitor, Rp-8-Br-PET-cGMPS (Supplementary Fig. S3). To extend these *in-vitro* findings to the *in-vivo* level, we studied sGC expression and activity in PKG knockout mice (PKG^−/−^)^33^. Consistent with our *in-vitro* data, sGC protein levels (Fig. 2D) and sGC activity (Fig. 2E) were unchanged in PKG^−/−^ compared to wildtype mice.

In conclusion, both our *in-vivo* and *in-vitro* data suggested that the downregulation of sGC protein and activity by chronic NO is cGMP- and PKG-independent and thus appeared to be due to a non-canonical mechanism (Fig. 2C). At least two cGMP-independent effects on sGC have been reported, rapid desensitization^10,20,34^, which is reversible in a thiol-dependent manner^35,36^, and oxidative heme-loss yielding the NO-insensitive apo-form of sGC (apo-sGC)^7,37^. These possibilities were tested in our two next sets of experiments.

### N-acetyl-L-cysteine does not prevent NO-induced sGC downregulation

**Figure 3:**
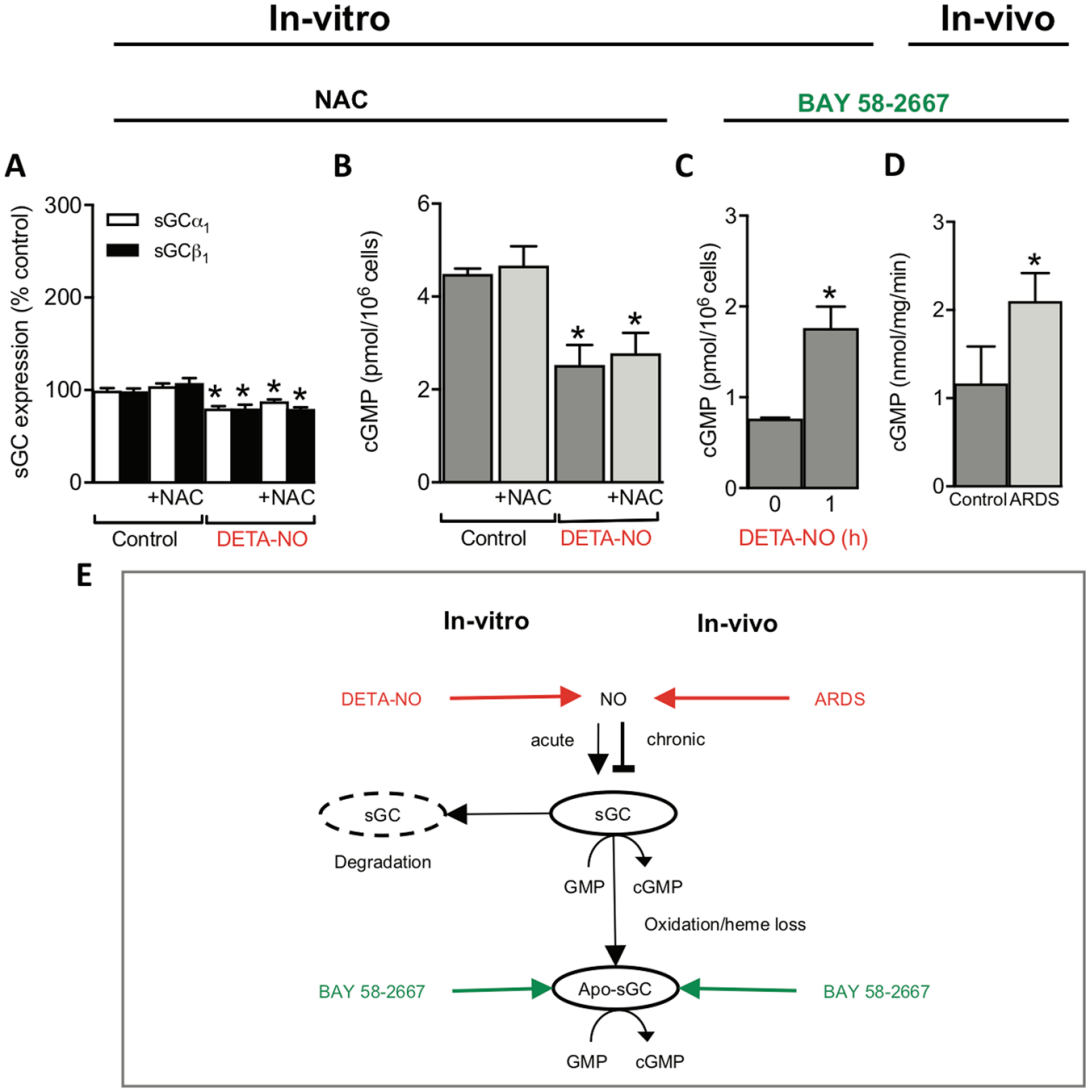
NO-induced sGC downregulation is thiol-independent but involves sGC loss and a shift towards apo-sGC. When PPAECs were exposed for 72 h to DETA-NO (100 µM) in the absence and presence of N-acetyl-L-cysteine (NAC; 1 mM), NAC neither affected sGC protein levels (N = 5) (**A**) nor activity (N = 4) (**B**). Exposure of PPAECs for 72 h to DETA-NO (100 µM) increased apo-sGC activity, measured as BAY 58-2667-induced cGMP formation (BAY 58-2667, 10 µM) (N = 3) (**C**). Validation of the above *in-vitro* mechanistic findings *in-vivo* in the porcine high-NO ARDS model showing also increased apo-sGC activity (N = 3) **(D)**. **(E)** A scheme summarizing both our *in-vitro* and *in-vivo* data that both endogenous NO or pharmacological NO donor compounds that acutely stimulate sGC, chronically decreased both sGC protein and activity leading to inactivation of sGC and an apparent net shift towards NO-insensitive apo-sGC. Data are expressed as mean ± SEM. *,**p < 0.05 or 0.01 *vs*. control, respectively. Representative full-length blots are presented in Supplementary Figure S4.

Thiol-sensitive mechanisms are involved in both sGC regulation, such as sGC maturation, and airway pathologies such as asthma^20^. Therefore, we assessed whether NO-posttranslational modification of free-thiol cysteines i.e., S-nitrosylation, contributes to the downregulation of sGC by high chronic NO incubation. For this approach, PPAECs were again exposed for 72 h to DETA-NO (100 µM) in absence or presence, over the full-time frame, of the membrane-permeable thiol-reducing agent, N-acetyl-L-cysteine (NAC; 1 mM). NAC is a membrane-permeable de-nitrosylating agent and glutathione precursor that has been extensively validated to protect sGC from nitrosylation^38,39^ down to concentrations as low as 1 mM^40,41^, which we, however, did not re-validate. NAC, however, neither affected sGC protein levels (Fig. 3A) nor sGC activity (Fig. 3B), suggesting that it is unlikely that a thiol-reversible mechanism similar to the acute desensitization is involved in the chronic NO-induced downregulation of sGC. These findings left oxidative heme-loss yielding apo-sGC^7,37^ as the most likely cGMP-independent effect on sGC.

### NO-induced sGC downregulation generates NO-insensitive sGC

We, therefore, examined whether chronic NO converts sGC to apo-sGC. To assay for the presence of apo-sGC, we took advantage of the apo-sGC activator, BAY 58-2667 (cinaciguat), which specifically binds to the empty heme-binding pocket of apo-sGC and re-activates cGMP formation in a NO-independent manner^42^. Indeed, up to 72 h exposure of PPAECs to DETA-NO (100 µM) increased apo-sGC activity, measured as BAY 58-2667-induced cGMP formation (Fig. 3C), and reduced sGC activity (Fig. 1D). To validate this mechanistic finding *in-vivo*, we re-examined the high-NO porcine ARDS model and found indeed apo-sGC activity to be increased (Fig. 3D). Collectively, this established apo-sGC formation as one possible mechanism of NO-induced reduction in sGC activity in addition to the loss in sGC protein (Fig. 3E).

## Discussion

Our findings close important gaps in our understanding of NO-cGMP signaling, in particular on the long-term effects of endogenously formed NO versus NO donors on sGC and the pathophysiological relevance of chronic NO for sGC regulation. Thus, we expand the previously observed notion that NO donor drugs can reduce sGC mRNA^43^ to the protein level and, importantly, from pharmacology to endogenous NO. Previously, sGC protein levels were not consistently investigated or with antibodies of unclear specificity^12,44^. Moreover, only in some cases were the effects of PKG on cGMP levels investigated^43,45^ or in relation to cGMP metabolism rather than cGMP formation^43,46,47^.

Surprisingly, not only pathological/high levels of NO, as in our porcine ARDS model but already low chronic endogenous NOS activity, suppressed sGC protein and activity in an L-NAME reversible manner. These findings establish a previously not recognized delicate steady state in the interactions between NO and sGC, acutely stimulating and chronically limiting its expression and activity. On a positive note, under conditions of diminished NO synthesis, this may, in turn, rapidly upregulate sGC protein and activity, as we have observed in the presence of the NOS inhibitor, L-NAME, and *in-vivo* in eNOS^−/−^ mice. In this regard, previous data are controversial. For example, sGC activity was increased in eNOS^−/−^ mice^13,48^, which agrees with our findings, while others found neither sGC expression nor activity to be changed, neither in eNOS^−/−^ mice^49,50^, nor upon treatment with high doses of NO donors^51^. The reasons for this discrepancy are unclear. In our ARDS model, the pan-NOS inhibitor, L-NAME, could not differentiate between eNOS and other isoforms present, such as iNOS^52^. Activation of the latter, e.g., by nuclear factor kappa B (*NFkB)*^53,54^, increases peroxynitrite leading to sGC oxidation^55^ and downregulation of sGCβ1^56^. In endothelial cells and under physiological conditions, however, iNOS is not expressed or only at low levels^57^ and thus unlikely to be involved in the here observed non-canonical feedback.

Therapeutically relevant is the previously not recognized risk of chronic use of NO donor drugs as they will lead to a downregulation of both sGC protein and activity. Together with their problematic pharmacokinetic tolerance^58–60^, this adds to the clinical limitations of this widely used drug class. With the introduction of NO-independent sGC stimulators and cGMP elevating agents into clinical practice^61^, there is now an alternative. Indeed, we show that the prototypic sGC stimulator and PDE inhibitor, YC-1, does not lead to sGC downregulation.

Concerning the underlying mechanisms, we initially considered two known mechanisms in NO-cGMP physiology, *i*.*e*., cGMP/PKG and thiol modification^9,10^. Surprisingly, both could be excluded, which was reminiscent of earlier observations where long-term exposure to an exogenous NO donor reduced sGC activity in a manner that could not be reversed or prevented with thiol treatment^14,46^. Instead, our findings suggest that endogenous and exogenous NO chronically induce a net shift from sGC to apo-sGC and that this is not only a pathophysiological mechanism but pertains to NO-cGMP physiology. Our results explain why apo-sGC activator-induced cGMP formation and functional effects are enhanced in disease but not exclusive to these conditions^7^. Nevertheless, the availability of sGC activator compounds allows now to overcome such conditions in which sGC protein and activity are diminished in favor of apo-sGC and still induce cGMP formation. As a limitation, other potential underlying mechanisms such as decreased mRNA^43^, increased degradation of sGC, or high NO-induced *NFkB* activation that downregulate sGC^56,62^ have not been addressed by our study^63^ and cannot be excluded.

Our findings also add to our understanding of apo-sGC as a therapeutic target. Hitherto apo-sGC has been mainly studied by using the heme oxidant, ODQ, or by expressing enzyme where the proximal heme ligating histidine had been deleted^64^. The mechanisms by which apo-sGC forms in pathophysiology were less clear. Now chronic exposure to (high) levels of NO can be considered as one of these conditions. There are at least three possible non-canonical mechanisms by which high levels of NO can induce the transition from sGC to apo-sGC. First, NO can interact with reactive oxygen species to form the potent oxidant, peroxynitrite^65–68^. Second, NO can impair heme synthesis^69^ or activate heme oxygenase-1 (HO-1)^70,71^, which increases sGC heme degradation^72^. However, this effect is controversial since high concentrations of NO donors (including DETA-NO) inhibit the heme degradation in endothelial cells^73^. Third, high NO can increase the association of sGCβ1 with heat shock protein 90 (Hsp90), but not with sGCα1, resulting in the formation of NO-insensitive sGC^20^.

Of note, the shift from sGC to apo-sGC is not 1-to-1. Some sGC appears to be lost due to inactivation beyond recovery by apo-sGC activators, e.g., by channeling into the ubiquitylation-proteasome pathway^74^. Nevertheless, an apparent net shift from sGC to apo-sGC as the primary source of cGMP formation is a common denominator and has recently been observed by us in another high NO model of ischemic stroke^6^ and others in an asthma model^20^. In contrast to other observations, in our settings, chronic NO incubation for 72 h versus overnight^20^, did affect sGC β1 expression independent of S-nitrosylation.

In conclusion, our data suggest that both *in-vitro* and *in-vivo*, and both under physiological conditions and in disease, NO self-limits its ability to induce cGMP formation via chemical redox feedback, which inactivates sGC and causes an apparent net shift towards apo-sGC. Our findings are of direct therapeutic importance as a pathological sGC/apo-sGC ratio can be treated with sGC activator compounds^74^, thereby reinstalling cGMP synthesis and PKG signaling^7,37^. Moreover, concerning the long-established class of NO donor drugs and the use of inhaled NO, a cautionary note needs to be added. Not only do they cause reversible tolerance, but also, as we now find, irreversible downregulation of sGC and apo-sGC formation. Our data explain, thus, also the superiority of the novel NO-independent sGC stimulators, at least in indications such as pulmonary hypertension^15^.

## Materials and Methods

### Chemicals

Polyclonal antibodies specific for sGCα_1_ and sGCβ_1_ have been described elsewhere (30). Actin monoclonal antibody (Oncogene Research Products, Boston, USA); collagenase type CLS II (Merck, Netherlands); 8-Bromo-cGMP (BIOLOG, Germany); L-NAME, DETA/NO, DEA/NO, IBMX and GTP (Enzo Life Sciences, Netherlands); BAY 58-2667 was synthesized as described^75^. All other chemicals were of the highest purity grade available and obtained from Sigma or Merck (Netherlands). DETA/NO and DEA/NO were dissolved in 10 mM NaOH, BAY 58-2667 and YC-1 in DMSO.

### Tissue isolation

Thoracic aortae from i) 6- to 8-months old male PKG^−/−^ and age-matched control mice were obtained from Prof. Franz Hofmann, Department of Pharmacology and Toxicology at the Technical University Munich (genetic background 129/Sv)^33^, and ii) 6- to 8-months old male eNOS^−/−^ mice and age-matched control were obtained from the Department of Physiology at Heinrich-Heine-Universität Düsseldorf (genetic background C57BL/6)^36^. Animals’ care was in accordance with guidelines of Technical University Munich and Heinrich-Heine-Universität Düsseldorf. Experimental protocols were approved by the animal ethics committees of Technical University Munich and Heinrich-Heine-Universität Düsseldorf. Thoracic aortae were grounded in a mortar to a powder that was used for protein determination, Western blots, or sGC activity assays. About 50 mg of tissue powder was suspended in homogenization buffer and shortly homogenized in an Ultra Turrax at 4 °C. Samples were diluted with four volumes of Rotiload buffer (Roth, Germany) and boiled for 10 min at 95 °C, centrifuged for 2–3 min at 14,000 rpm, and the supernatant used for protein determination and protein immunoblot analysis.

### Preparation of pulmonary arteries from a porcine ARDS model

Pigs were acclimated for at least 24 h before use in the study and handled carefully to avoid any stress. Body temperature was kept constant using a circulating-water heating pad and cage heating. The porcine ARDS model was induced as previously described^76^. Briefly, pigs (30–35 kg) were pre-medicated with midazolam (1 mg/kg i.m.) and ketamine (10 mg/kg i.m.); intravenous anesthesia was induced and maintained with midazolam (bolus 0.5–1 mg/kg, followed by 1–2 mg/kg/h) and ketamine (bolus 3–4 mg/kg, followed by 10–18 mg/kg/h). Neuromuscular block was achieved with atracurium (bolus 3–4 mg/kg, followed by 1–2 mg/kg/h). Pigs were mechanically ventilated in a volume-controlled mode with the following settings: Tidal volume (V_T_) of 8 ml/kg, positive end-expiratory pressure (PEEP) of 5 cm H_2_O, the fraction of inspired oxygen (FIO_2_) of 1.0, respiratory rate (RR) adjusted in accordance to an arterial partial pressure of carbon dioxide (PaCO_2_) between 35–45 mmHg and inspiration to expiration (I:E) of 1:1. Following this preparation, acute lung injury (ALI) was established according to a two-hit model^76–79^. After established ALI, a lung-protective ventilation strategy was initiated with the following settings: pressure control ventilation (PCV), V_T_ of 6 ml/kg, PEEP of 16 cm H_2_O, FIO_2_ of 0.5, RR adjusted in accordance to a PaCO_2_ between 35-45 mmHg and I:E of 1:1. Mild hypovolemia was induced by means of drainage of 25% of circulating blood volume. After resuscitation with different colloids (HAES 6%, 130/0.4, and gelatin 4%) and crystalloid solutions (Ringer Acetate, Baxter), the lung-protective ventilation was maintained for 4 h. The overall duration of mechanical ventilation was 10-11 h. After that, animals were sacrificed by intravenous boluses of 2 g thiopental and 50 ml KCl 1 M, and organs were snap-frozen in liquid nitrogen. Pulmonary arteries were removed immediately after death, snap-frozen in liquid nitrogen and stored at minus 80 °C or otherwise processed immediately to tissue powder and subsequently suspended in homogenization-buffer and homogenized in an Ultra Turrax at 4 °C. These samples were then used further for protein determination, protein immune blots, and sGC activity assays.

### PPAECs

Fresh porcine pulmonary arteries were obtained from a local slaughterhouse and maintained in phosphate-buffered saline (PBS; 10 mM Na_2_HPO_4_, 1.8 mM KH_2_PO_4_, 140 mM NaCl, 2.7 mM KCl, pH 7.4) at 37 °C. PPAECs were isolated enzymatically by incubation of the aorta inner surface with collagenase type CLS II (0.5 mg/mL for 10 min at room temperature) and then collected in HEPES-buffered medium 199. After centrifugation (250 × g, 10 min) the pellet was re-suspended in growth medium (medium 199 supplemented with 10% fetal calf serum, 100 U/mL penicillin, 100 µg/mL streptomycin) and cells were propagated in coated plastic flasks and incubated (37 °C, 6% CO_2_). Upon confluence, endothelial cell monolayers were sub-cultured in 35-mm (for Western blot) or 60-mm (for cGMP determination) gelatin-coated dishes. Confluent cell monolayers from the second passage were used for experiments. PPAECs consistently formed a viable confluent monolayer without signs of significant cell loss, e.g., cell detachment or clumping. In all pilot experiments and intermittent controls, no cell death occurred during the 72 h exposure to 100 µM DETA/NO consistent with previous publications^80–82^. The growth medium was replaced either every 12 or 24 hours if applicable containing the indicated compounds. After incubation time, cells were subsequently used for sGC activity measurements or western blot analysis.

### Detection and quantification of sGC protein

Western blotting procedures were described previously^83^. Briefly, cells were lysed in 250 µL Roti-Load sample buffer (ROTH, Karlsruhe, Germany), preheated to 95 °C and then boiled for an additional 10 min before loading on SDS gel electrophoresis. Primary antibodies were diluted 1:4000 for anti-sGCα_1_ and 1:2000 for anti-sGCβ_1_ antibody in 3% dry milk in TBST and incubated with nitrocellulose membranes at 4 °C over-night following challenge of membranes with secondary goat anti-rabbit antibody (1:2000 in 3% milk in TBST) conjugated to horseradish peroxidase (Dako A/S, Denmark). Immuno-complexes were visualized using an enhanced chemiluminescence kit (Amersham Pharmacia Biotech, Freiburg). Samples were quantified with a Kodak Imager Station 440 CF and with the NIH 1.6 software, and all blots were standardized to ß-actin or GAPDH expression that was not affected by the treatments. Representative western blot examples are shown in Supplementary Fig. S4.

### Measurement of cGMP levels

For measuring cGMP levels, cells were pretreated for 30 min with the phosphodiesterase inhibitors^84^, IBMX (1 mM) and zaprinast (100 μM). Then, sGC was consistently stimulated with 250 µM DEA/NO or 10 µM BAY 58-2667 for 3 min at 37 °C. The high concentration of DEA/NO (250 µM) was chosen based on previous studies using endothelial cells, including PPAECs^63,67,85^. However, other studies showed that cGMP accumulation in endothelial cells in response to DEA/NO peaked at about 1 µM^86^. Of note, however, there are controversial data regarding the NO peak concentration released from DEA/NO. One study^87^ showed that 250 µM DEA/NO would give a peak NO concentration of about 30 µM while another study^88^ showed that 1 mM DEA/NO would release 2.87 μM/min of NO during 3 minutes. Therefore, 250 µM would release 0.71 μM/min of NO. Additional factors, such as medium components may be responsible for this broad range of apparent free concentrations of NO.

After sGC stimulation, cells were immediately lysed in 80% ethanol. Cells were scraped and, after evaporation of ethanol, resuspended in assay buffer and sonicated. Measurement of sGC activity in crude homogenates of porcine tissue was performed as previously described^83^. Briefly, all samples were measured as the formation of cGMP at 37 °C during 10 min in a total incubation volume of 100 µl containing 50 mM triethanolamine-HCl (pH 7.4), 3 mM MgCl_2_, 3 mM glutathione, 1 mM IBMX, 100 mM zaprinast, 5 mM creatine phosphate, 0.25 mg/ml creatine kinase and 1 mM or 0.5 mM GTP. The reaction was started by the simultaneous addition of the sample and either DEA/NO or BAY 58-2667, respectively. After incubation of each sample for 10 min, the reaction was stopped by boiling for 10 min at 95 °C. Thereafter the amount of cGMP was subsequently determined by a commercial enzyme immunoassay kit (Enzo Life Sciences, Netherlands).

### Statistics

For comparisons, Student’s t-test or multiple comparisons one-way analysis of variance (ANOVA) was followed by Bonferroni’s test. Calculations were performed using GraphPad Prism 6.0 (GraphPad Software, San Diego, USA). All data are expressed as mean ± SEM. P-value <0.05 was considered significant.

## Supplementary information

Supplementary information.

## Data Availability

All data needed to evaluate the conclusions in the paper are present in the paper or the Supplementary Materials.
