# Supplementary material for: Non-canonical chemical feedback self-limits nitric oxide-cyclic GMP signaling in health and disease
Source: Sci Rep. 2020 Jun 19;10:10012. doi: 10.1038/s41598-020-66639-w (PMC7305106; doi:10.1038/s41598-020-66639-w)
Supplement: Supplementary file 1 — Supplementary information. [file 41598_2020_66639_MOESM1_ESM.pdf]

Supplementary information

Non-canonical chemical feedback self-limits nitric oxide-cyclic GMP signaling in health and disease

Vu Thao-Vi Dao[1,2], Mahmoud H. Elbatreek[1,3*], Martin Deile[4], Pavel I. Nedvetsky[5], Andreas Güldner[6], César Ibarra-Alvarado[7], Axel Gödecke[8] and Harald H.H.W. Schmidt[1*]

[1]Department of Pharmacology and Personalised Medicine, MeHNS, FHML, Maastricht University, Maastricht, The Netherlands;

[2]Department of Pharmacology, Johannes Gutenberg University, Mainz, Germany

[3]Department for Pharmacology and Toxicology, Faculty of Pharmacy, Zagazig University, Zagazig, Egypt;

[4]Asklepios-Klinik Radeberg, Dresden, Germany;

[5]Universitätsklinikum Münster, Medical Cell Biology, Münster, Germany; nedvetsk@uni-muenster.de

[6] Residency Anaesthesiology, Department of Anaesthesiology und Critical Care Medicine, Technische Universität, Dresden, Germany

[7] Facultad de Química, Universidad Autónoma de Querétaro, Mexico

[8] Institut für Herz- und Kreislaufphysiologie Heinrich-Heine-Universität, Düsseldorf, Germany

*Corresponding Author: Prof Harald Schmidt, Department for Pharmacology and Personalised Medicine, MeHNS, FHML, Maastricht University, PO Box 616, 6200MD Maastricht, the Netherlands, Phone: +61-3-9905-5752; Email: hschmidt@ppmlab.net and melbatreek@ppmlab.net

Key words: endothelial cells; soluble guanylate cyclase; nitric oxide; cyclic guanosine mono phosphate; redox regulation; Bay 58-2667, apo-sGC

SUPPLEMENTARY FIGURES

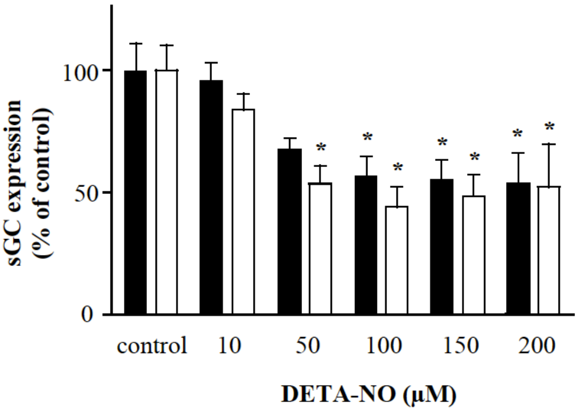

Supplementary Fig. S1. 100µM DETA-NO is the maximal concentration to downregulate sGC. Treatment of PPAECs for 72h with different concentrations of DETA-NO showed that 100µM is the maximal concentration to downregulate both sGCα$_1$ (solid bars) and sGCβ$_1$ (open bars) (N=6). *P< 0.05 by one-way analysis of variance (ANOVA), data represent means ± SEM.

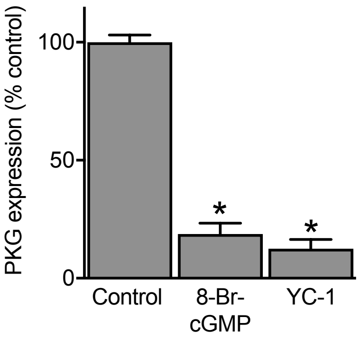

Supplementary Fig. S2. PPAECs display intact NO-cGMP-PKG signaling since treatment with 8-Br-cGMP (500 µM), or YC-1 for 72hrs reduced PKG expression (N=6-9). *P< 0.05 by one-way analysis of variance (ANOVA), data represent means ± SEM.

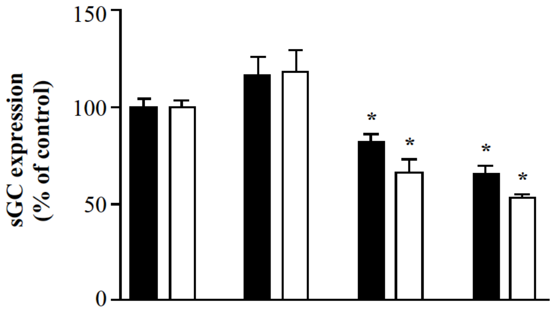

100 µM DETA-NO	-	-	+	+
10 µM Rp-8-Br-PET cGMPS	-	+	-	+

Supplementary Fig. S3. Inhibition of PKG does not prevent DETA-NO induced downregulation of sGCα$_1$ and sGC β$_1$. Treatment of PPAECs for 72h with either 100 µM DETA-NO, or with the PKG inhibitor Rp-8-Br-PET-cGMPS (10 µM), or a combination thereof resulted in down regulation of sGCα$_1$ (solid bars) and sGCβ$_1$ (open bars) level that could not be reversed by Rp-8-Br-PET-cGMPS (N=6). *P< 0.05 by one-way analysis of variance (ANOVA), data represent means ± SEM.

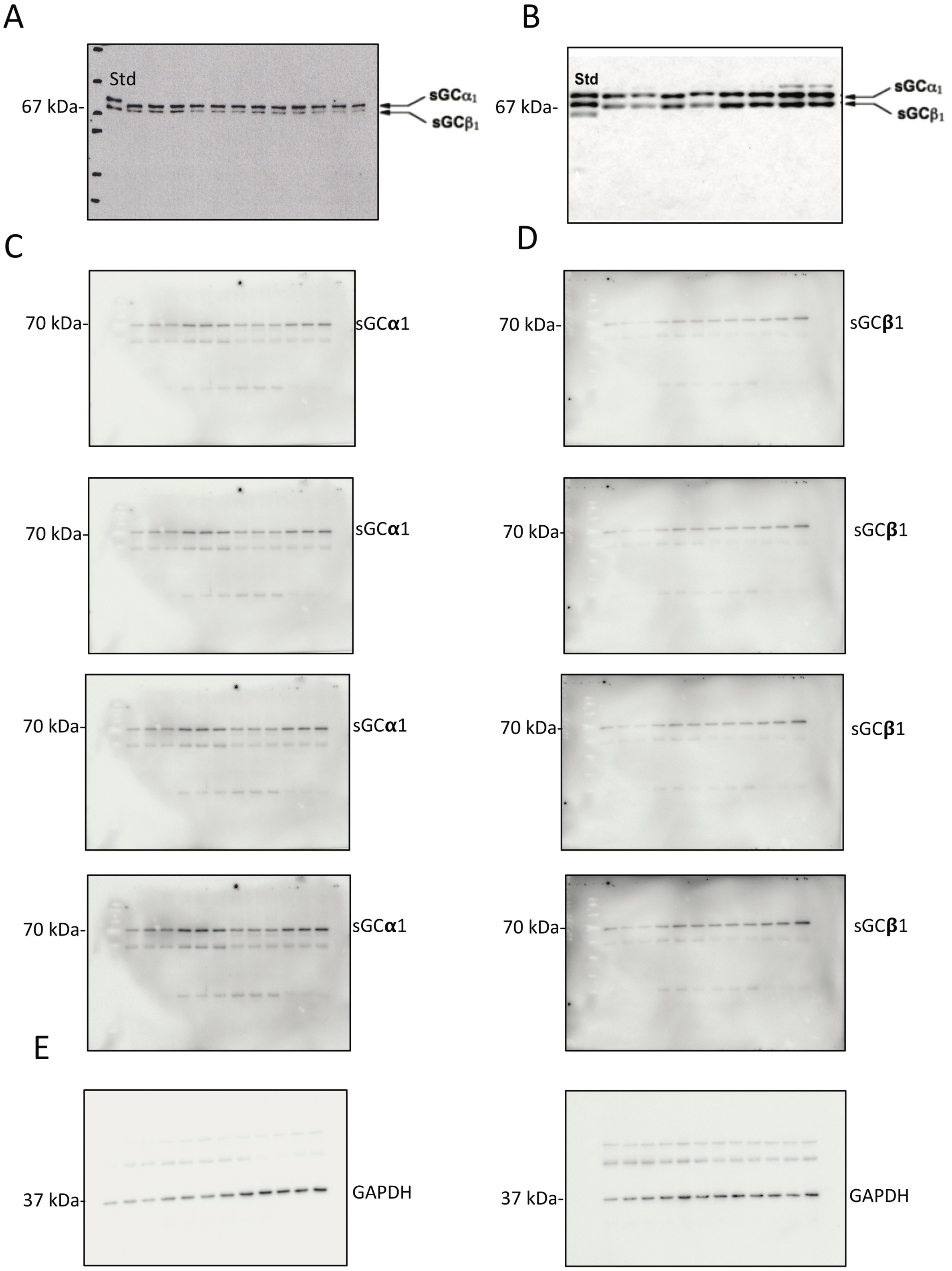

Supplementary Fig. S4. Representative Immune detection blots of controls, sGCα_1 and sGCβ_1. A. PPAECs. B. Mouse. C-E. ARDS (Multiple exposures).
